# Studying Acoustic Behavior of BFRP Laminated Composite in Dual-Chamber Muffler Application Using Deep Learning Algorithm

**DOI:** 10.3390/ma15228071

**Published:** 2022-11-15

**Authors:** Wael A. Altabey, Mohammad Noori, Zhishen Wu, Mohamed A. Al-Moghazy, Sallam A. Kouritem

**Affiliations:** 1 International Institute for Urban Systems Engineering (IIUSE), Southeast University, Nanjing 210096, China; 2 Department of Mechanical Engineering, Faculty of Engineering, Alexandria University, Alexandria 21544, Egypt; 3 Department of Mechanical Engineering, California Polytechnic State University, San Luis Obispo, CA 93405, USA; 4 School of Civil Engineering, University of Leeds, Leeds LS2 9JT, UK

**Keywords:** acoustic characteristics, laminated composite, BFRP, deep learning, dual-chamber muffler

## Abstract

Over the last two decades, several experimental and numerical studies have been performed in order to investigate the acoustic behavior of different muffler materials. However, there is a problem in which it is necessary to perform large, important, time-consuming calculations particularly if the muffler was made from advanced materials such as composite materials. Therefore, this work focused on developing the concept of the indirect dual-chamber muffler made from a basalt fiber reinforced polymer (BFRP) laminated composite, which is a monitoring system that uses a deep learning algorithm to predict the acoustic behavior of the muffler material in order to save effort and time on muffler design optimization. Two types of deep neural networks (DNNs) architectures are developed in Python. The first DNN is called a recurrent neural network with long short-term memory blocks (RNN-LSTM), where the other is called a convolutional neural network (CNN). First, a dual-chamber laminated composite muffler (DCLCM) model is developed in MATLAB to provide the acoustic behavior datasets of mufflers such as acoustic transmission loss (TL) and the power transmission coefficient (PTC). The model training parameters are optimized by using Bayesian genetic algorithms (BGA) optimization. The acoustic results from the proposed method are compared with available experimental results in literature, thus validating the accuracy and reliability of the proposed technique. The results indicate that the present approach is efficient and significantly reduced the time and effort to select the muffler material and optimal design, where both models CNN and RNN-LSTM achieved accuracy above 90% on the test and validation dataset. This work will reinforce the mufflers’ industrials, and its design may one day be equipped with deep learning based algorithms.

## 1. Introduction

**Figure 1:**
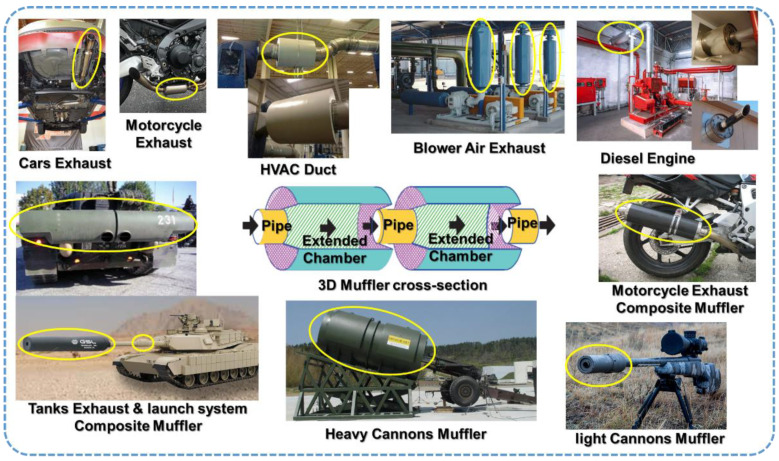
Examples of the Industrial and Military equipment which are connected with mufflers.

A muffler is considered as a common device for passive noise elimination. This device is widely used in various equipment and machines which are connected with ducts or exhaust systems, such as diesel engines, HVAC system, compressors, blowers and fans for ventilation [1]. The laminated composite muffler is also used in military equipment and machineries such as tanks, heavy, and light cannons, due to the unique thermo-acoustic and mechanical characteristics of composite materials (see Figure 1). However, mufflers are often limited by space availability. Consequently, there has been rising interest in designing mufflers to optimize acoustic characteristics. There are several optimized TL methods for the optimization of muffler shape under space constraints that have been developed by several researchers over the last two decades [2,3,4]. Numerous studies have also been carried out by many researchers on mufflers to estimate the noise attenuation performance. These studies have suggested various evaluation methods for the actual noise reduction in mufflers when an optimally designed muffler is mounted on a duct [5,6,7].

### 1.1. Related Work

Since the focus of this research is on the application of laminated composite mufflers, the literature on acoustic behavior of composite materials had to also be investigated. Presently, research on the acoustic behavior of composite materials is becoming increasingly mature. This is largely due to good dynamic and mechanical properties, especially the high strength/lightweight properties, of these types of materials. Numerous studies have been carried out on composite materials to estimate the acoustic behavior using both experimental [8,9] and numerical prediction methods [10,11], while the conclusions have shown that the acoustic behavior of composite laminated mufflers need to be further investigated in order to develop a better understanding of the acoustic performance of these types of mufflers.

#### 1.1.1. Acoustic Transmission Loss (TL)

Transmission loss (TL) generally describes the cumulative degradations in the acoustic power transmission of a waveform as a wave propagates out a source, or as it propagates through a given area or through a particular type of structure such as mufflers. It is a frequently used terminology in acoustics and is measured in decibels. TL measurements are very important in the industry of acoustic devices such as mufflers and sonars.

To improve the TL accuracy of mufflers, several simulation studies have been reported on how to obtain an optimized shape for mufflers. 

Numerical methods such as the finite element method (FEM) [12] and the boundary element method (BEM) [13,14] are suitable for predicting the TL performance of reactive and dissipative mufflers and are not limited to the geometry method of mufflers. The radiation impedance form of an unflanged circular pipe was developed by Levine and Schwinger [15], and another form of radiation impedance procedure was suggested for an infinitely large flanged circular pipe by Norris and Sheng [16]. For a radiation impedance formula, Silva et al. [17] presented approximate mathematical expressions for a complicated end shape. The radiation impedance and the reflection coefficient relationship for a circular pipe was presented by Polack et al. [18]. The radiation impedance measurement methods were summarized and calculated for several boundary conditions by Dalmont et al. [19,20].

#### 1.1.2. Mufflers Design Optimization

In most muffler design problems for sound reduction, the muffler has been designed in two separate parts, the main pipe or duct and the muffler part. The TL in the muffler has been widely used for evaluating the muffler performance during the optimization process of the muffler size [21,22,23]. However, the optimal muffler performance of the sound attenuation may be degraded during the installation process due to the long distance between the main pipe and the radiation impedance. The length of the main pipe mounted on the muffler is usually longer than the length of the outlet of a muffler. These problems motivated the authors to find an effective method to optimize the muffler size in order to improve the evaluation of the sound attenuation performance in a muffler. Lee et al. [24] studied developing the multi-chamber muffler with dissipative elements to reduce TL of noise. They used FEM and experimental methods to design a muffler that consists of dual expansion connected in a series of flow systems that effectively optimize sound in the speech interference range [25]. Lee et al. [26] proposed a multi-objective topology optimization problem to maximize the TL at the target frequency while minimizing the voltage drop. Shi et al. [27] studied experimentally and theoretically the propagation of sound wave in a periodic array pipe mufflers with micro-holes. They found that the periodic structure affected the performance of the muffler with micro-holes. Du et al. [28] studied experimentally the acoustic performance of water muffler based on a Kevlar-reinforced rubber tube for reducing the noise by optimizing vibration damping and the hydrodynamic noise reduction method.

#### 1.1.3. Deep Neural Networks (DNNs)

The artificial neuronal network (ANN) is a mathematical model of information-processing established by imitating the structure and function of the brain neuronal network. This method implies repeated learning and training of known information, and includes a scheme to gradually adjust the learning weights of neurons to imitate the relationship between the input and output data. Compared to the traditional method, the ANN technique has obvious advantages in the processing of blurred data, random data and nonlinear data, and is especially suitable for complex large-scale structures and unclear information systems. 

DNN is an ANN architecture with multiple hidden layers. Depth generally refers to the amount of hidden layers in the structure of the DNN. Learning is a cognitive process of the unknown to the known, corresponding to the input and output process in the structure of the DNN. Deep learning strengthens the learning ability of feature and makes the prediction more accurately through the hierarchical expression of the input information and the transformation of present layer feature by a previous layer.

### 1.2. Contribution 

Generally, mufflers reduce the sound pressure generated by sound-generating sources such as motors, fans, etc. existing in systems of a vehicle exhaust or the home ventilation by generating dissipative sound waves caused by geometric discontinuity by means of acoustic impedance difference. Therefore, internal partitions should be optimally placed to improve the noise attenuation performance in the main noise frequency range when the outer size of the muffler is limited. Muffler material is one of the important parameters in sound insulation and absorption. The most methods used for evaluating the acoustical performance of a muffler (TL) are the FEM, traditional laboratory methods, four-pole transfer matrix, and the three-point methods.

**Figure 2:**
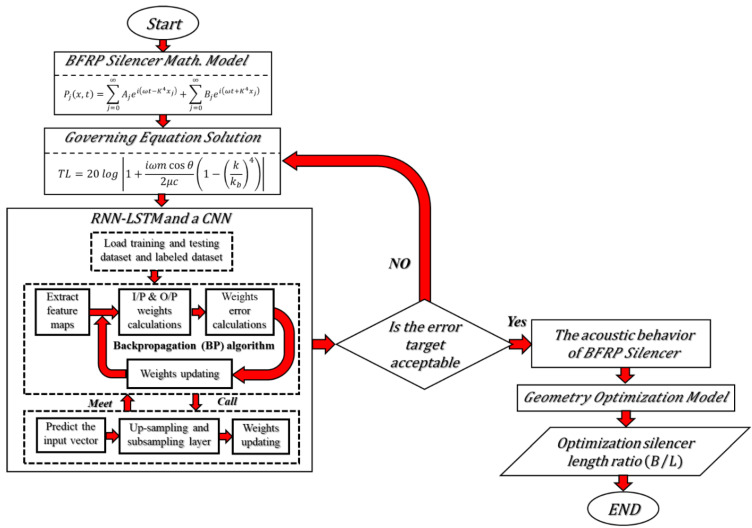
Flowchart of the proposed method.

The presented work utilizes two types of ANN architectures which are called RNN-LSTM and CNN. These two types of ANNs are developed in Python in order to predict the acoustic behavior of a DCLCM manufactured from a BFRP laminated composite. Figure 2 shows a flowchart of the proposed method. The PTC and the TL of an acoustic muffler will be calculated from the exact solution of the governing acoustic equations of the muffler model. After the essential characteristics of the muffler have been extracted, the muffler characteristics are fed to an ANN as labeled data. The inputs and outputs weights of the network are calculated. Then, we compute the derivative of the error for weights by the backpropagation (BP) algorithm. If the error target is not acceptable “case no”, the muffler characteristics data are fed again to an ANN to train and calculate new weights to compute the new error and so on until the error target is acceptable “case yes”. The training and model parameters were optimized by using BGA Optimization. The proposed work will be discussed more in depth in the following sections.

In this work, to develop a monitoring strategy for the acoustic behavior performance of DCLCM, a novel deep learning algorithm is utilized. The muffler acoustic performance is calculated for a wide range of geometric data of the DCLCM using commercial software. An RNN-LSTM and a CNN are trained, evaluated, and developed via Python. BGA optimization is used to optimize the training and the parameter selection for the model. To the best of the authors’ knowledge, the methodology presented in this paper and the results they obtained is an original contribution to scientific research for monitoring an acoustic behavior in composite muffler materials.

To achieve the goals presented above, this work is arranged as follows: Section 2 presents system modeling and discusses a mathematical model including case studies for composite muffler systems. Results and discussion are presented in Section 3, including an algorithm established and proposed utilizing a deep neural network. Conclusions are presented in Section 4, and a brief summary as well as a plan of future work are reported in Section 5.

## 2. Methods and Materials

### 2.1. Case Study

**Figure 3:**
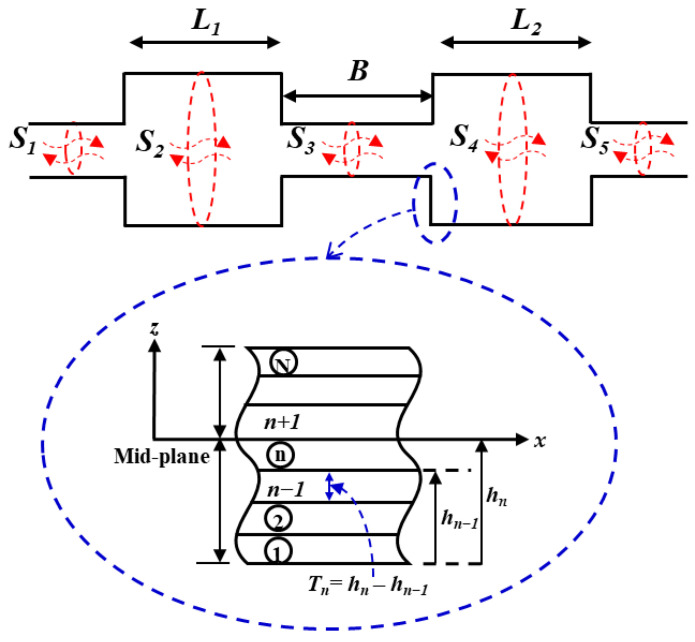
The geometrical model of the DCLCM.

Figure 3 shows the geometry of a circular DCLCM considered in this study. An acoustic DCLCM was constructed from a BFRP laminated composite, the staking distribution to three symmetrically plies is [0/90°/0]_s_, and the thickness of each ply is 5 mm. The BFRP Physical and mechanical properties are shown in Table 1. The geometric information used for the calculations in this work is summarized in Table 2. The values of the muffler thickness, B, to the total chamber length, L, are listed in Table 2. The range of frequencies used for PTCs calculation of muffler is [0–3 KHz]. The muffler length, B, is optimized in order to maximize the acoustic TL. At the right end of the muffler, an anechoic termination is assumed.

Table 1 shows the physical and mechanical properties of the BFRP, where ρ is the material density, E_11_, E_22_ are elastic modulus in the ‘1’and ‘2’ directions, respectively, G_12_, G_21_ are the shear modulus, υ_21_, υ_12_ are the Poisson’s ratio of the transverse strain in the directions ‘1’ and ‘2’ caused by the normal stress in the directions ‘2’ and ‘1’, respectively.

#### 2.1.1. Basic Acoustic Equations of the Dual-Chamber Muffler

**Figure 4:**
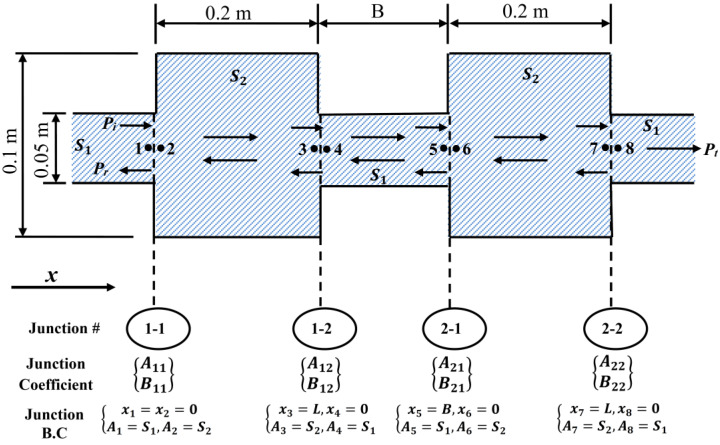
The mathematical space constraints for DCLCM.

Figure 4 presents the mathematical model of DCLCM which consists of three straight pipes and two expansion chamber pipes being identified. As shown in the figure,$\;S_{1}=S_{3}=S_{5}$ is the area of the straight pipe, and $S_{2}=S_{4}$ is the area of the muffler. Eight points were chosen to represent the flow condition inside the muffler (pt_1_~pt_8_). At each two consecutive points, the standing pressure $\;P_{s}$ and reflected $\;P_{r}$ pressure are similar e.g.,$\;P_{s_{1}}+P_{r_{1}}=P_{s_{2}}+P_{r_{2}}$; in addition, the standing $U_{s}$ volume velocity and reflected $U_{r}$ volume velocity are similar e.g., $U_{s_{1}}+U_{r_{1}}=U_{s_{2}}+U_{r_{2}}$. The continuity algorithm of pressure and volume velocity on continuity junctions numbers $\left(1-1\right),\;\left(1-2\right),\;\left(2-1\right),\;\left(2-2\right)$ are applied to compute junctions’ transmission coefficients $A_{11},\;B_{11},A_{12},\;B_{12},\;A_{21},\;B_{21},\;A_{22},\;B_{22}$, by solving the Helmholtz Equation (1) [30], and applying the junction boundary condition (B.C) at each junction:

(1) $$\nabla^{2}P+k^{2}P=0$$

For the equal size chamber systems shown in Figure 4, the three-point method was used for describing acoustical properties via absorbing materials assuming notation of the plane wave propagation since the transverse system dimensions are smaller than one wavelength over the frequency range of interest. A standing pressure propagating wave amplitude $A_{j}$ and a reflected pressure propagating wave amplitude $B_{j}$ are assumed. The standing $\;P_{s}$ and reflected $\;P_{r}$ pressure wave can be expressed in Equations (2) and (3) respectively as:

(2) $$\;P_{s}=\overset{\infty }{\underset{j=0}{\sum }}A_{j}e^{i\left(\omega t-K^{4}x_{j}\right)}$$

(3) $$P_{r}=\overset{\infty }{\underset{j=0}{\sum }}B_{j}e^{i\left(\omega t+K^{4}x_{j}\right)}$$

where j is the junction number, $K=\frac{k}{k_{b}}$ is wavenumber ratio, k=ω/c is the wavenumber, ω  is the angular frequency, c=330[m/s] is the sound speed in air, and $k_{b}$ is the bending wavenumber of the laminated composite pipe.

#### 2.1.2. Acoustic Properties of Composite Laminated Muffler

The laminated muffler studied in this paper is made of BFRP composites in general. Due to the low strength and stiffness characteristics in the transverse direction of laminate, it does not consist only of the unidirectional lamina. Thus, some laminas in most laminates are placed at an angle. Therefore, the stress–strain relationship for an angle lamina must be developed. The new axis is called local axes in the 1–2 coordinate system, where direction 1 is parallel to the fibers, and direction 2 is perpendicular to the fibers. The angle between the local axes in the 1–2 coordinate system and the global axes in the *x*–*y* coordinate system is the fiber angle θ. The plane stress transformed reduced stiffness coefficients ${\bar{Q}}_{ij}$ of the lamina can be expressed in terms of the engineering notations (see Table 1) as:

(4) $$Q_{ij}=\left[\begin{array}{ccc} Q_{11} & Q_{12} & Q_{13}\\ Q_{12} & Q_{22} & Q_{23}\\ Q_{13} & Q_{23} & Q_{66} \end{array}\right]=\left[\begin{array}{ccc} \frac{E_{11}}{\left(1-\upsilon_{12}\upsilon_{21}\right)} & \frac{\upsilon_{21}E_{11}}{\left(1-\upsilon_{12}\upsilon_{21}\right)} & 0\\ \frac{\upsilon_{21}E_{11}}{\left(1-\upsilon_{12}\upsilon_{21}\right)} & \frac{E_{22}}{\left(1-\upsilon_{21}\upsilon_{12}\right)} & 0\\ 0 & 0 & G_{12} \end{array}\right],$$

(5) $${\bar{Q}}_{11}=Q_{11}\cos^{4}\theta +2(Q_{12}+2Q_{66})\sin^{2}\theta \cos^{2}\theta +Q_{22}\sin^{4}\theta$$

(6) $${\bar{Q}}_{12}=(Q_{11}+Q_{22}-4Q_{66})\sin^{2}\theta \cos^{2}\theta +Q_{12}(\sin^{4}\theta +\cos^{4}\theta )$$

(7) $${\bar{Q}}_{22}=Q_{11}\sin^{4}\theta +2(Q_{12}+2Q_{66})\sin^{2}\theta \cos^{2}\theta +Q_{22}\cos^{4}\theta$$

(8) $${\bar{Q}}_{66}=(Q_{11}+Q_{22}-2Q_{12}-2Q_{66})\sin^{2}\theta \cos^{2}\theta +Q_{66}(\sin^{4}\theta +\cos^{4}\theta )$$

The bending stiffness $D_{ij}$ can be calculated from:

(9) $$D_{ij}=\frac{1}{3}\sum_{n=1}^{N}{\left[\left({\bar{Q}}_{ij}\right)\right]}_{n}(h_{n}^{3}-h_{n-1}^{3}),\;\;\;\;i\;j\;=\;1,\;2,\;3,\ldots \ldots$$

Using the above analysis of composite material, it can be found that the muffler performance can be greatly affected by the laminating plies staking and fiber angle *θ*. When the muffler is excited by sound waves, there is a relationship between the sound pressure inside such a DCLCM and the normal vibration velocity [31].

#### 2.1.3. Acoustic Transmission Loss (TL)

In mufflers, the theoretical definition of TL is the logarithmic ratio of incident to power transmission for the case of reflection-free terminations. This can be expressed in terms of sound pressure by solving Equation (10):

(10) $$P_{j}\left(x,t\right)=\overset{\infty }{\underset{j=0}{\sum }}A_{j}e^{i\left(\omega t-K^{4}x_{j}\right)}+\overset{\infty }{\underset{j=0}{\sum }}B_{j}e^{i\left(\omega t+K^{4}x_{j}\right)}$$

where $k_{b}$ is the bending wavenumber of the laminated composite pipe:

(11) $$k_{b}{}^{4}=\frac{m\omega^{2}}{sin^{4}\theta \left(D_{11}cos^{4}\theta +2\left(D_{12}+2D_{66}\right)sin^{2}\theta cos^{2}\theta +D_{22}sin^{4}\theta \right)}\;\;\;\;\;\;\;\;$$

where $m=\frac{S_{2}}{S_{1}}$ is the area ratio.

**Figure 5:**
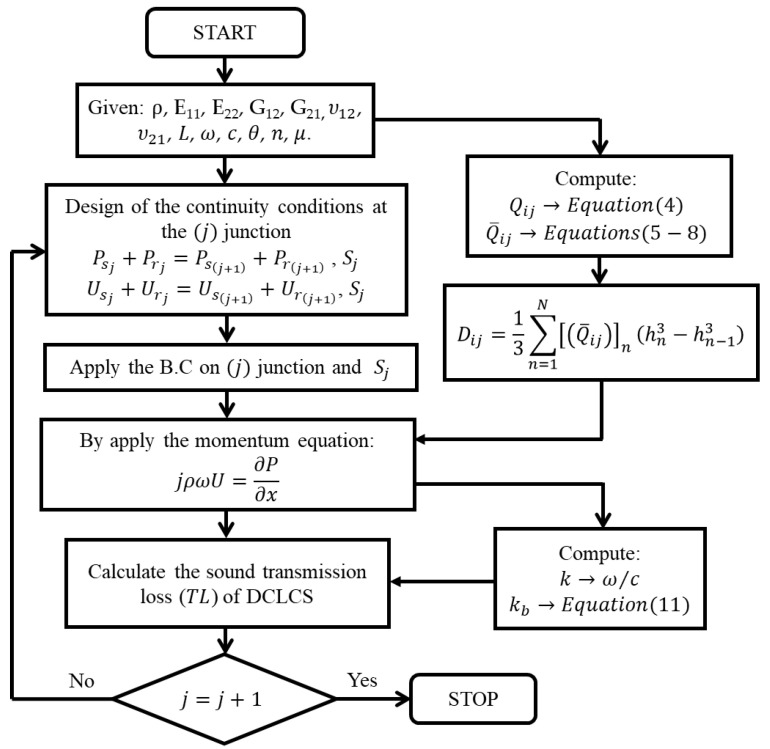
Algorithm of the governing equation solution technique to find the muffler TL.

Figure 5 presents the algorithm flow chart of the Equation (10) solution technique to find the acoustic pressure $P_{j}$ wave in each junction (j) of the DCLCM model (see Figure 4). By applying algorithms of continuity of pressure (P) in Equation (12) and volume velocity (U) in Equation (13) and substituting with boundary conditions (B.C) at each junction (j) in the DCLCM model based on the flow condition inside the muffler (pt_1_~pt_8_) and section area $\left(S_{j}\right)\;$(see Figure 4):

(12) $$P_{s_{j}}+P_{r_{j}}=P_{s_{\left(j+1\right)}}+P_{r_{\left(j+1\right)}},\;{\left(B.C\right)}_{j}\;based\;on\;\left(pt_{1}\sim \;pt_{8}\right),\;S_{j}$$

(13) $$U_{s_{j}}+U_{r_{j}}=U_{s_{\left(j+1\right)}}+U_{r_{\left(j+1\right)}},\;\;{\left(B.C\right)}_{j}\;based\;on\;\left(pt_{1}\sim \;pt_{8}\right)\;,\;S_{j}$$

where j is the junction number, and U denotes the axial acoustic velocity which can be obtained by the momentum equation:

(14) $$i\rho \omega U=\frac{\partial P}{\partial x}$$

where ρ is the fluid density, and $i=\sqrt{-1}$ is the imaginary unit. Then, the eigenfunctions’ orthogonal properties are used [32], where $L_{1}=L_{2}=L$ and $S_{1}=S_{3}=S_{5}$,$\;S_{2}=S_{4}$, and the junction boundary condition (B.C) is applied. Consequently, the resultant sound TL of DCLCM model resulted in being:

(15) $$TL=20\;log\left|1+\frac{i\omega m\cos\theta }{2\rho c}\left(1-{\left(\frac{k}{k_{b}}\right)}^{4}\right)\right|$$

### 2.2. Artificial Neural Networks 

ANN is the most used algorithm between different artificial intelligence (AI) algorithms for the advanced nonlinear problems solution [33,34]. Each individual network of ANN consists of the number of computational nodes, and each node is used for processing the inputs and transferring the input calculation results to output connections. Each node output may be an input to another node or more. Weights and biases are used to scale and bias the outputs, respectively, e.g., in the function *y = mx + b*, *y* and *x* refer to output and new output, respectively, *m* is the weight, and *b* is the bias. Some networks are activated to determine the output with the type of function being linear or non-linear by adding activation functions. The rectified linear unit (ReLU) is the activation function most used in deep learning [35]. 

Before adjusting the weights and biases in ANN, the model does not work well, i.e., the ANN model is not trained. The learning of NN can be done automatically from raw data as a hierarchical feature representation [36,37] or can be trained via case study examples. In our models designed in this work, we used supervised learning to train, by comparing the training data and model’s predictions to actual data. The training accuracy can be improved via updating the trainable parameters to optimize the error between prediction and actual data. 

ANN can be determined depending on the type of input data, such as the case study in this work; the ANN models are used to analyze the time-series data.

In this work, the two major ANN model types for analyzing the time series data that were discussed are the RNN-LSTM and CNN.

#### 2.2.1. Convolutional Neural Network (CNN)

CNNs are used to analyze the groups of data such as the time series, images, sentences, sound recordings, etc. Weight matrices in CNNs are applied as kernels or filters to extract the features [38,39].

**Figure 6:**
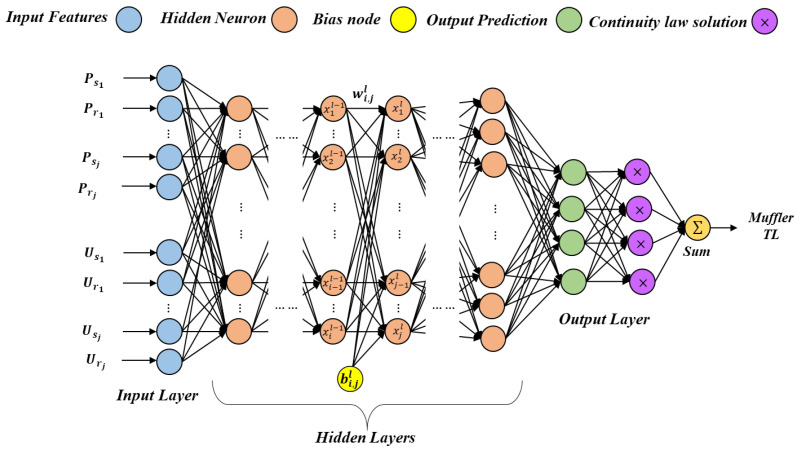
An architecture of proposed CNN with a fully connected network.

As shown in Figure 6, a proposed CNN model typically consists of feature extraction through a stack of layers on the input layer such as convolution, activation and poling, and classification through fully connected layers for outputting the scores for each class. Each layer is responsible for different functions and uses the result from the previous layer as the input.

Equation (16) describes the operation of proposed CNN, and this process is defined as:

(16) $$x_{j}^{l}=f\left(\underset{i}{\sum }x_{i}^{l-1}w_{ij}^{l}+b_{j}^{l}\right)$$

where $x_{j}^{l}$ is the *ith* output map in layer l;$\;x_{i}^{l-1}$ is the *ith* output map in layer l−1;$\;w_{ij}^{l}$ is the weight; $b_{j}^{l}$ is the bias; $f\left(\cdot \right)$ is a nonlinear function that is applied component-wise.

#### 2.2.2. Recurrent Neural Network with Long Short-Term Memory Blocks (RNN-LSTM)

**Figure 7:**
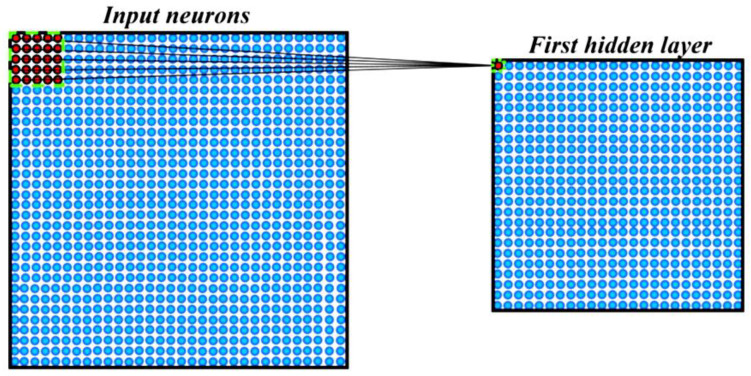
A 5 × 5 filter rolling around an input volume and generating an output.

RNNs deeply analyze the time series data via applying the feedback loops to original ANN [40]. The biggest disadvantage in RNNs is known as the vanishing gradient problem, where, during the backpropagation process, the error signal used to train the network exponentially decreases the further you travel backwards in RNN, thus sometimes using computational nodes known as LSTM to relieve this problem, as shown in Figure 7. The data feature extraction is done from the first layers of ANN. These layers are responsible for extracting significant information from the input data [41].

**Figure 8:**
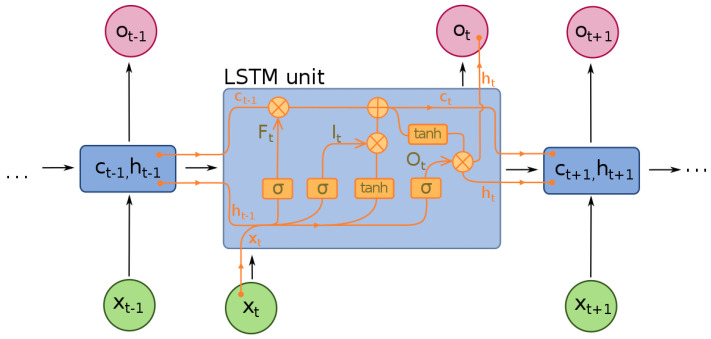
A single block diagram in an RNN-LSTM [41].

LSTM is a special type of RNN with gating mechanism and memory cells, which greatly improves the performance of RNNs. There are three types of gates within each LSTM cell: input gate, forget gate, and output gate, and these gates define the state of each memory cell by using sigmoid as the activation function to cause information to be transmitted selectively. The memory cell that retains the long-term state $c_{t}$ is the key architecture of each LSTM cell. The internal architecture of a single LSTM cell is shown in Figure 8.

We can describe the operation of the three gates presented in Figure 8 in Equations (17)–(19). Equations (20)–(22) suggest the cell states $c_{t}$ and the hidden state $h_{t}$ of each LSTM unit at time t:

(17) $$i_{t}=\sigma \left(W_{i}\cdot \left[h_{t-1},x_{t}\right]+b_{i}\right)$$

(18) $$f_{t}=\sigma \left(W_{f}\cdot \left[h_{t-1},x_{t}\right]+b_{f}\right)$$

(19) $$o_{t}=\sigma \left(W_{o}\cdot \left[h_{t-1},x_{t}\right]+b_{o}\right)$$

(20) $${\overset{\prime }{c}}_{t}=tanh\left(W_{c}\cdot \left[h_{t-1},x_{t}\right]+b_{c}\right)$$

(21) $$c_{t}=f_{t}⨀c_{t-1}+i_{t}⨀{\overset{\prime }{c}}_{t}$$

(22) $$h_{t}=o_{t}⨀\tanh\left(c_{t}\right)$$

where $W_{f}$,$\;W_{i}$,$\;W_{c}$, and $W_{o}$ represent the weight matrices of LSTM; $b_{f}$,$\;b_{i}$,$\;b_{c}$, and $b_{o}$ denote the bias vector of LSTM;$\;f_{t}$,$\;i_{t}$, and $o_{t}$ are forget gate, input gate, and output gate vectors at time t;$\;c_{t-1}$ and ${\overset{\prime }{c}}_{t}$ mean, respectively, the previous cell state and a new candidate value.$\;\sigma \left(z\right)$ and $tanh\left(z\right)$ are utilized as the activation functions, as shown below:

(23) $$\sigma \left(z\right)=\frac{1}{1+e^{-z}}$$

(24) $$tanh\left(z\right)=\frac{e^{z}-e^{-z}}{e^{z}+e^{-z}}$$

#### 2.2.3. Bayesian Genetic Algorithms (BGA) Optimization

**Figure 9:**
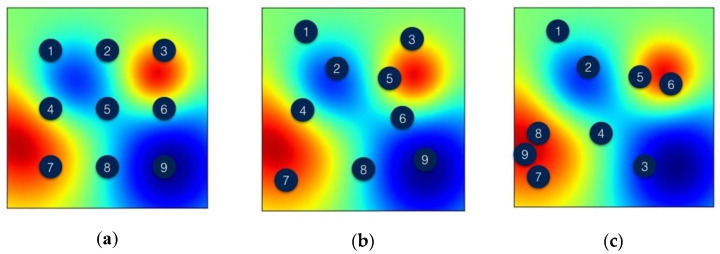
The comparison between (**a**) Grid Search vs. (**b**) Random Search vs. (**c**) BGA Optimization techniques for tuning the model’s hyperparameters.

Generally, there are several strategies for BGA optimization modeling of objective functions (*f*), such as Gaussian processes [42,43,44], random forests [41], and tree-structured Parzen estimators [45,46]. Figure 9 presents the comparison between the Grid search and BGA Optimization tuning method for tuning the model’s hyperparameters. As shown in the figure, the yellow dot refers to the assessment of the model in each method; note that the grid search method may be searched in rough space, but the BGA optimization methods can test any possible combination in space and intelligently suggest combinations to obtain optimal solutions with fewer evaluations. In this research, we applied the BGA techniques for quick, intelligent tuning.

#### 2.2.4. The Work Description

With the desire to explore alternative indirect Monitoring Acoustic Behavior of DCLCM frameworks and inspired by deep learning, this work discusses the framework of deep learning that analyzes TL data of DCLCM to optimize the acoustic TL of the DCLCM. To evaluate the idea of the presented work, the work was divided into the following sections:

(1) **Data Collecting:** The ability and accuracy of the designed ANN are based on the volume of feeding data to the ANN; this accuracy will be improved when there are more data for training. In the present work, the data were computed via MATLAB from a DCLCM model. This dataset was used for training, validation, and test for the ANN.
(2) **The Established Algorithm:** from the data collected in the previous step, via Python, an RNN-LSTM and CNN are developed and then, using BGA, we are training parameters and tuning models.

**Figure 10:**
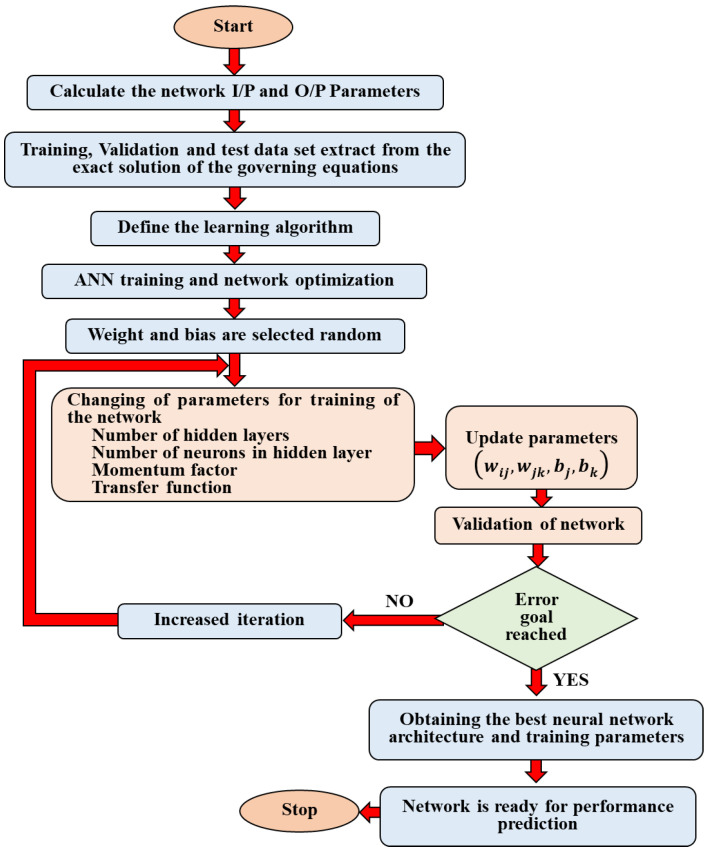
The flowchart of the ANN model framework.

Figure 10 presents the flowchart of framework for the proposed ANNs used in this work. As shown in Figure 10, the weight coefficients between the processing neurons are adjusted to reach some desired goal during the training process. The neurons are interconnected via feed-forward considered links for the basic neuron computation. The multiplication results of the neuron input and the connection weight between input layer and hidden layer are obtained. Then, a bias value is added to them using Equation (16). The results of calculation processes are subjected to an activation function. Activation function generates neuron output results. Similar operations can be applied to the output layer. The activation function determines the input/output behaviors of the network. The error, known as the difference between the ANN output and desired output, is calculated. Finally, the new weight values are obtained. This method must be repeated until the error is acceptable. This training method is continued for all the data in the training dataset.

## 3. Results and Discussion

### 3.1. Validation of the Proposed Method

**Figure 11:**
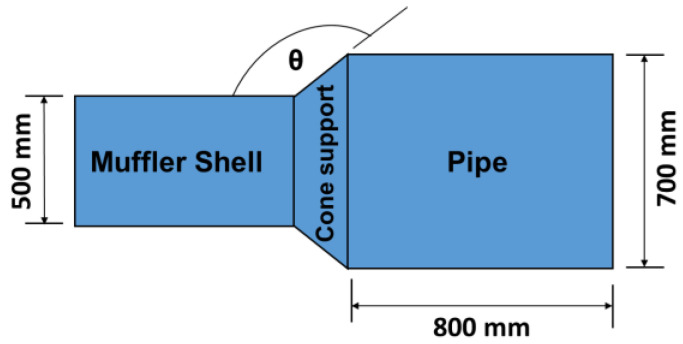
The muffler structure.

In this subsection, a convergence investigation is carried out for the proposed method. The acoustic TL at all octave Band Center Frequencies (OBCFs) are calculated and compared with available experimental results in literature. Table 3 presents a convergence and comparison study for a muffler structure optimization design to eliminate the noise in the pumping system by Liu et al. [47]. The muffler is silenced at the outlet of the pump pipeline system, the muffler material is commercial steel and the structure of muffler is shown in Figure 11. 

**Figure 12:**
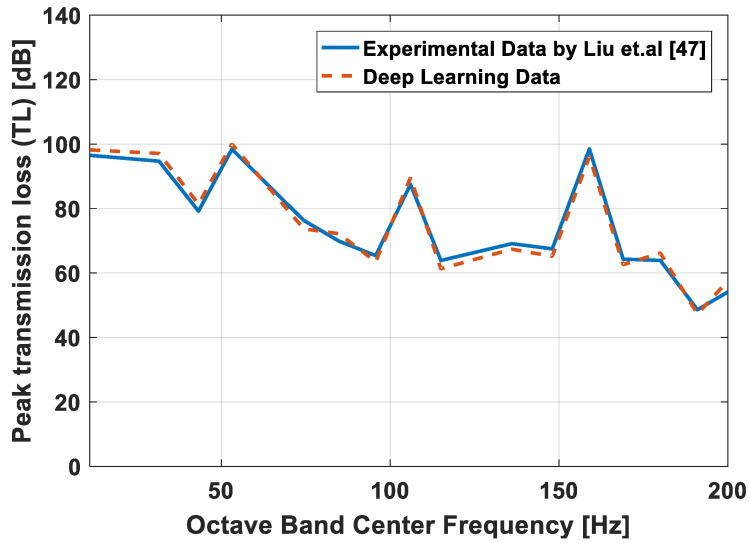
Comparison between the proposed method and experimental values of Peak transmission loss (TL) of the muffler.

The experimental test device used by Liu consists of a booster pump, motor, valve, pressure transmitter, flowmeter, muffler, data acquisition system, etc. They studied the noise reduction performance by using three values for the expansion angle of the flow channel (θ) which are 120°, 145°, and 160°. They found that, when the extension angle was 145°, the muffler had the best sound attenuation effect. In this validation, the measured data of the experiment for muffler with extension angle 145° were compared with the data of the proposed method simulation data. As shown in Table 3 and Figure 12, we can see the acoustic TL at all OBCFs that were obtained by test of the muffler had the same trend as that of the proposed method calculation.

### 3.2. Data Collecting

In this work, the dimensions of the DCLCM model and all S/C ratios (B/L) that were used in the most calculations in the analytical section are included in Table 2.

**Figure 13:**
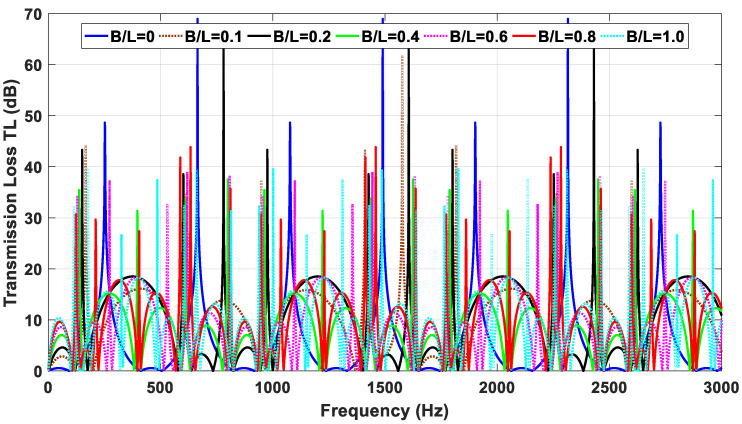
The TL of DCLCM with different S/C ratio (B/L).

**Figure 14:**
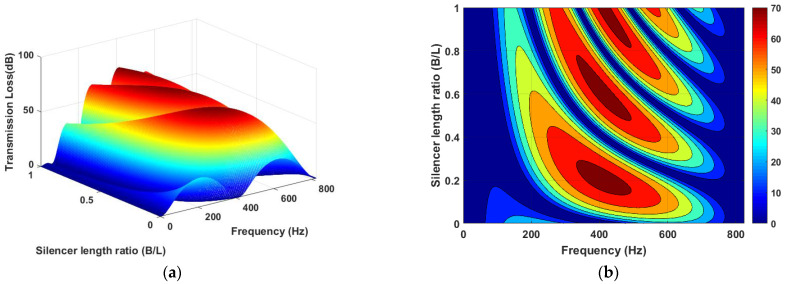
Modeled TL vs. S/C ratio (B/L) and full periodicity of frequency (**a**) 3D plot and (**b**) contour plot.

A MATLAB model for the DCLCM representation as shown in Figure 3 was run at various S/C ratios to generate simulated examples of DCLCM system response. The simulation solves the system acoustic equations to find acoustic pressure in each junction of the DCLCM for every pressure and volume velocity at each junction in the DCLCM system. The simulation inputs are extracted from below Figure 13 and Figure 14 and their accompanying derivations are presented in Equations (9)–(30).

The acoustic TL properties for the proposed muffler are illustrated in Figure 12 and Figure 13 (for *B/L* = 0, 1.0, 0.2, 0.4, 0.6, 0.8, 1.0), where high attenuation is evident over a wide frequency range. Figure 13 presents the acoustic TL distribution over all OBCFs. 

The DCLCM behavior is presented in Figure 13, where the acoustic TL distribution changes over the frequency range with the change in muffler dimensions. Therefore, we find that the expansion chamber without a muffler (S/C ratio equal zero) has equal domes, and the higher the S/C ratio, the more it changes the domes to an unequal shape, and the the first dome has the smaller amplitude and frequency band than another one at each muffler geometrical configuration in S/C ratios presented in Table 2. The rate of rise and fall of domes increases the acoustic TL with the increase in frequency so that it reaches a peak at a certain frequency that then decreases and so on. In addition, the S/C ratio (B/L) in the muffler geometry has an effective effect in sound attenuation of TL value, and we find that the increases in the S/C ratio (B/L) have an effect on widening of the second dome and tend to cover two domes or more of the acoustic TL for the lower S/C ratios. The cut-off frequency on which the behavior of acoustic TL operates is 3000 Hz. At all S/C ratios (B/L) presented in Table 2, the muffler effect on the TL value has almost sequentially vanished at frequencies 130, 615, 825, 1440, 1780, 2270, and 2600 Hz, respectively.

Figure 14 shows the acoustic TL distribution with respect to S/C ratio over one completed period of frequency from 0 to 825 Hz. As shown in Figure 14, at stationary frequency, the value of acoustic TL gain changes with the increases of S/C ratio (B/L), which indicates that the acoustic TL has a high sensitivity to S/C ratio (B/L) changes. Consequently, we can consider that the S/C ratio is the main parameter of frequency shifting, and that it is the powerful key in the acoustic TL gain.

### 3.3. The Established Algorithm 

The establishing, training, and evaluating of a CNN and or RNN were done in Python software, and each neural network was developed via the same steps as listed in Algorithm 1 below based on steps discussed in Figure 10.

**Algorithm 1**. Training and evaluating of CNN and or RNN.1: **algorithm** CNN or RNN
2:          **input**: d: TL datasetl: S/C ratio (B/L)W: Network parameter matrix weight $w_{ij},w_{jk}$ and bias $b_{j},b_{k}$
3:       **output**: score of CNN or RNN trained model on test dataset to predict TL for various S/C ratio (B/L)
4:       **let** f be the feature set 3d matrix
5:       **for** i in dataset **do**
6:         **let**
$f_{i}$ be the feature set matrix of sample I
7:         **for** j **in** i **do**
8:            $v_{j}$ ← vectorize _(j, w)_
9:                **append**
$v_{j}$ to $f_{i}$
10:             **append**
$f_{i}$ to f
11:        $f_{\mathrm{train}}$,${\;f}_{\mathrm{test}}$,${\;l}_{\mathrm{train}}$,${\;l}_{\mathrm{test}}\;$← split feature set and prediction into train subset and test subset
12:            M ← CNN ($f_{\mathrm{train}}$,${\;l}_{\mathrm{train}}$) or RNN ($f_{\mathrm{train}}$,${\;l}_{\mathrm{train}}$)
13:            score ← evaluate (I,${\;l}_{\mathrm{test}}$, M)
14:            **return** score
15: **end for**
16: **end for**

### 3.4. ANN Models Development

**Figure 15:**
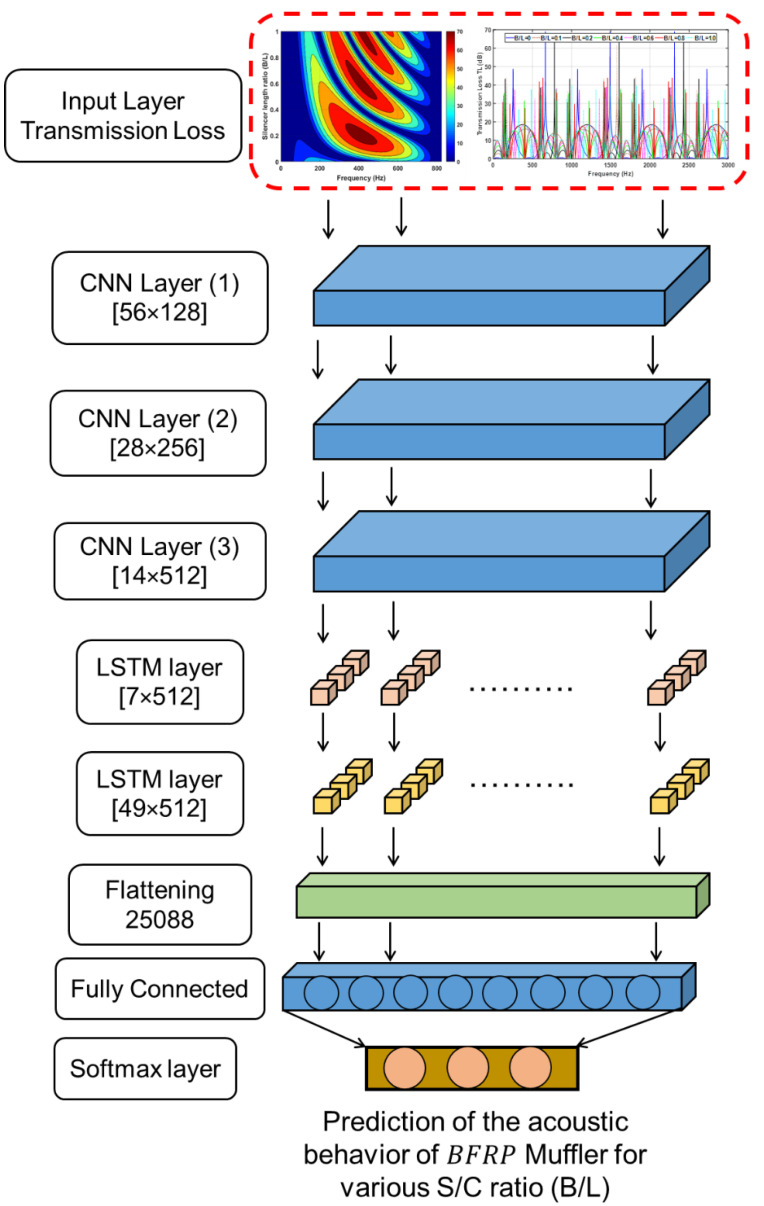
The three 1D convolutions’ full connection layer (FC) layers, two sub-sampling LSTM layers, and a softmax layer Deep Neural Network.

In this work, as shown in Figure 15, the deep learning neural network used mainly has an input layer of the muffler characteristics data, and three 1D convolutions’ full connection (FC) layers for training each layer are 56×128, 28×256, 14×512, respectively, for two sub-sampling LSTM layers, each layer is 14×512,  7×512, respectively, and then all the resultant 2D arrays from pooled feature maps are converted into a single long continuous linear vector in a flattening layer that has 25,088 elements in one linear vector, and a softmax layer as the activation function in the output layer that predicts a multinomial probability distribution of muffler characteristics such as TL datasets. In this work, both models of RNN and CNN have similar configurations, except the region close to the input layer for the feature extraction maps. The cost and accuracy of both models are evaluated, and the test set accuracy is also evaluated before and after training. The BGA technique was used to optimize the training speed and accuracy, respectively.

### 3.5. Final Results and Discussion

#### 3.5.1. Performance Evaluation of ANN Models

**Figure 16:**
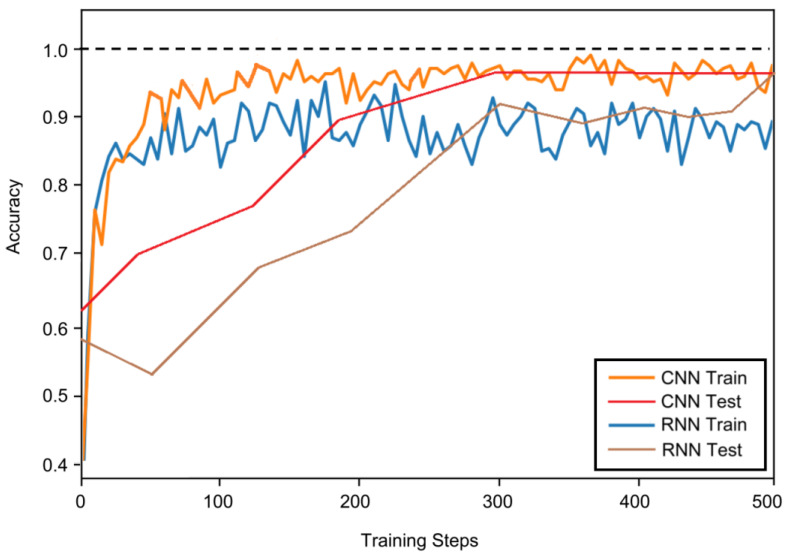
The ANN models’ sensitivity accuracy during training.

Figure 16 shows the final performances for both models. As shown in Figure 16 presenting accuracy curves during the training of training and validation datasets, both models CNN and RNN achieve accuracy above 90% on test and validation datasets, saving sufficient time. Furthermore, we also calculate the training and testing time of RNN-LSTM and CNN as shown in Table 4.

In addition, the performance of the CNN model is better than the RNN-LSTM model in classification ability. The additional computations are what distinguish the performance of CNN over RNN-LSTM. These computations are for feeding the hidden layer from the previous step in order to provide long-range contextual information into the next step. This means that the RNN-LSTM is incorrectly based on the built-up memory. On the other hand, the CNN can train and classify input data quickly and accurately because the CNN extracts the features within windows of time including time-series data.

The drawbacks of the CNN in the application of Monitoring Acoustic Behavior of DCLCM are the same as any applications of time-based series. The CNN performance is based on the dataset size and quality, but, in the presented work, the dataset is small and clean from noisy. In addition, in general, the poor random overfitting problem is one of the CNNs’ weaknesses, but, in this work, this problem was not observed. As a result, for further understanding the weaknesses of the CNN, more work must be done using a more complex dataset. Simply, if directly compared against fundamental equations of the convolution layer, we can see the contrast in complexity. If the previous layer inputs have a large number of filters, the CNN model still easier and faster to train and more simple than the RNN-LSTM model.

#### 3.5.2. Acoustic DCLCM Geometry Design Optimization

For selecting the optimum DCLCM Geometry Design, a genetic optimization method is used. A genetic algorithm (GA) is a stochastic global search and optimization method that mimics the metaphor of natural biological evolution. This process leads to the evolution of populations of individuals that are better suited to their environment than the individuals from which they were created, just as in natural adaptation.

**Figure 17:**
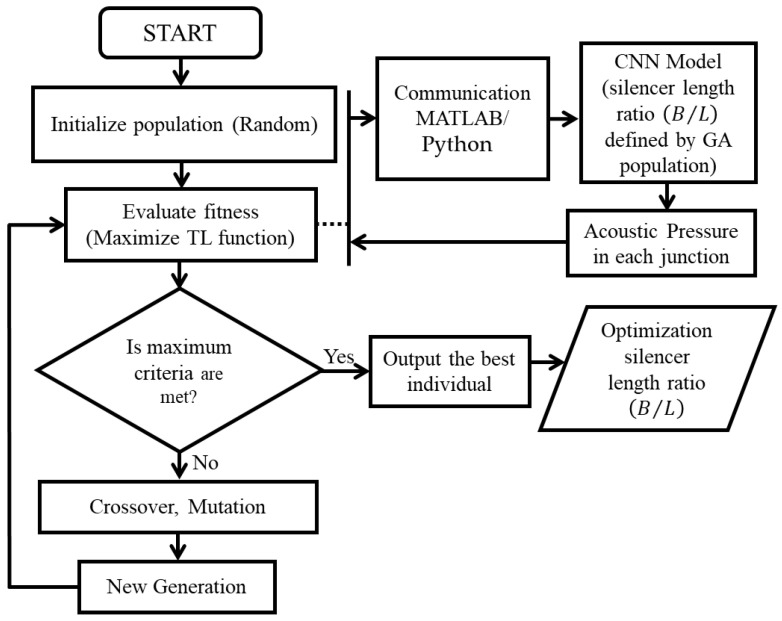
The flowchart of optimization methodology.

Figure 17 illustrates the flow chart of a genetic optimization method applied in this paper. In this case, the DCLCM junction locations as a string of integer numbers are encoded analogous to the genetic code on a DNA string; accordingly, the DCLCM acoustic outputs of TL are propagated over different OBCFs, and it is analogously identical to the behavior of the breeding population for a number of individuals, each characterized by its DNA, and each individual is determined according to some fitness function. Here, it is preferable to raise individuals with high fitness (high OBCFs) in the breeding process, so that useful genes tend to propagate through the generations (High OBCFs) and detrimental genes disappear (low OBCFs), just like with the range of acoustic frequencies generated by DCLCM. Usually, a GA code was written using MATLAB. 

As the muffler development depends on optimizing its geometry design for high performance, and because TL is an essential characteristic of the muffler, as well as the S/C ratio $\left(B/L\right)$ having a high sensitivity to acoustic TL, the $\left(B/L\right)$ is therefore a powerful parameter in the gain of acoustic TL. Actually, the $\left(B/L\right)$ is the key parameter for muffler development. 

**Figure 18:**
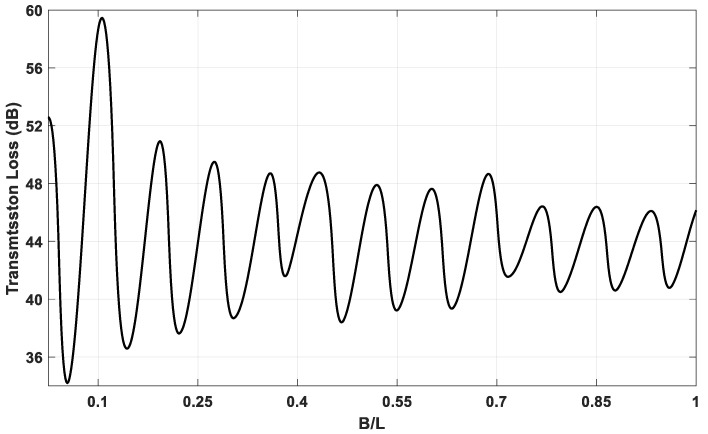
TL vs. S/C ratio (B/L).

**Figure 19:**
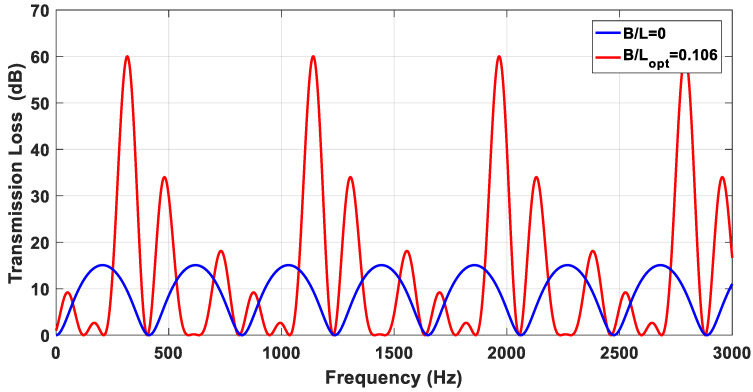
TL of DCLCM with optimal value of S/C ratio (B/L), compared with the system without muffler B/L=0.

In this section, we will use the generated simulated acoustic output of deep learning analysis of TL for acoustic TL maximization. The optimal value of the dimension (B) is planned and carried out. From the final performances for both models of ANNs used in this work, the performance of the CNN model is better than the RNN-LSTM model; therefore, we will use the acoustic TL output of the CNN model in the optimization processes. It should be noted that the derivation processes of the maximum value of the acoustic TL were achieved using MATLAB software. Figure 18 and Figure 19 show the results of these derivations in order to pick out the suitable dimension (B) which maximizes the value of TL.

Figure 19 shows the relationship between the values of TL and frequency range of DCLCM with optimal value of the S/C ratio (B/L), compared with the system without a muffler. As shown in Figure 19, the maximum value of TL can be obtained at the DCLCM resonance frequency (612 Hz), which is estimated analytically. Therefore, the maximum attenuation value will be 60 dB. It is possible to conclude that the optimization method is the better way to obtain the maximum value of TL corresponding to the defined dimensions range of DLCS. As a result of the DLCS dimensions optimization, the optimal value of S/C ratio (B/L) is 0.106. This value is corresponding to the higher-level sound attenuation of TL value.

## 4. Conclusions

This work studies the solutions of drawbacks of experimental and numerical works to study an acoustic behavior of different mufflers’ materials to improve its performance, where it is necessary to perform large, important, time-consuming calculations, particularly if the muffler is made from advanced materials such as composite materials. All of these problems were resolved with modern methods in this study by using a deep learning algorithm to predict the acoustic behavior of the muffler material in order to save effort and time on muffler design optimization. The acoustic behavior of DCLCM made from a BFRP laminated composite was predicted in Python through two types of DNN architectures which are RNN-LSTM and CNN. A comparison between these two architectures is presented in terms of the speed of training and accuracy of predictive data. We found that both the CNN and RNN-LSTM models achieved accuracy above 90% on test and validation datasets. In addition, the performance of the CNN model is better than the RNN-LSTM model in acoustic monitoring ability. The acoustic parameters of a transmission loss (TL) and the power transmission coefficient (PTC) were computed by solving the exact solution of the governing acoustic equations of the muffler model in MATLAB. The model training parameters are optimized by using Bayesian genetic algorithms (BGA) optimization. The acoustic TL output of the CNN model defined that the optimum muffler length is 0.0212 m with a fixed pipe radius and encountered the maximum acoustic TL of 60 dB in the resonance frequency of DCLCM (612 Hz). This work will reinforce the mufflers’ industrials in the future, and its design may one day be equipped with deep learning based algorithms.

## 5. Future Work

It will be interesting in the future to apply the proposed methodology in this work with some special boundary conditions and change the composite laminate stacking and filer angle or to add some special structures such as honeycomb materials between the consecutive stacking. Furthermore, a realistic experiment in the standing wave tube may make the results more convincing and useful. I hope that the study can not only give a basic reference to engineering applications but also inspire the researchers who are interested in composite laminates’ acoustic properties.

## Figures and Tables

**Table 1:** Physical and mechanical properties of the BFRP.

E_11_ (GPa)	E_22_ (GPa)	G_12_ (GPa)	G_21_ (GPa)	υ_12_	υ_21_	$\rho \;\mathrm{kg}/m^{3}$
96.74	22.55	10.64	8.73	0.3	0.6	2700

Note: This mechanical parameter property can be found in [29].

**Table 2:** The parameters’ specific values of the DCLCM model used for acoustical analysis.

Symbol	Description	Value (Unit)						
B/L	A muffler to chamber length (S/C) ratio	0	0.1	0.2	0.4	0.6	0.8	1.0
$R_{C}$	Chamber radius	0.1 (m)						
$R_{P}$	Inlet pipe radius	0.05 (m)						
L	Total chamber length	0.2 (m)						

**Table 3:** The peak transmission loss (TL) of the muffler expansion angle of the flow channel 145° along the frequency range.

Octave Band Center Frequency (OBCF) (Hz)	Expansion Angle of the Flow Channel 145°	
	Liu et al. [47]	Proposed Method
11.1	96.5	98.2
31.5	94.7	97.1
43.2	79.2	81.4
53.1	98.4	100
74.4	76.3	73.7
84.8	69.9	72.2
95.7	65.4	63.5
106	87.5	89.6
115	63.9	61.3
136	69.1	67.4
148	67.5	65.2
159	98.5	96.1
169	64.3	62.6
180	63.9	66.2
191	48.6	47.3
200	54.1	57.5

**Table 4:** Training time of RNN-LSTM and CNN.

Model	Training Time (Second)	Testing Time (Second)
RNN-LSTM	87.45	0.1
CNN	18.46	0.001

## Data Availability

Not applicable.

## Abbreviations

AI	Artificial intelligence.
ANNs	Artificial neural networks
B.C	Boundary conditions
BFRP	Basalt fiber reinforced polymer
BGA	Bayesian genetic algorithms
BP	Backpropagation
CNN	Convolutional neural network
DCLCM	Dual-chamber laminated composite muffler
FC	Full connection
I/P	Input
LSTM	Long short-term memory
OBCF	Octave band center frequency
O/P	Output
PTC	Power transmission coefficient
ReLU	Rectified linear unit
RNN	Recurrent neural network
RNN-LSTM	Recurrent neural network with long short-term memory
S/C	Muffler to chamber length
TL	Transmission loss

## References

[1] Tracor Inc. (1973). Guidelines on Noise.

[2] Guo R., Tang W. (2017). Transfer matrix methods for sound attenuation in resonators with perforated intruding inlets. Appl. Acoust..

[3] Yu X., Cheng L. (2015). Duct noise attenuation using reactive silencer with various internal configurations. Sound Vib..

[4] Oh K.S., Lee J.W. (2017). Topology optimization for enhancing the acoustical and thermal characteristics of acoustic devices simultaneously. Sound Vib..

[5] Lee J.W., Jang G.W. (2012). Topology design of reactive mufflers for enhancing their acoustic attenuation performance and flow characteristics simultaneously. Int. J. Numer. Methods Eng..

[6] Lee J.W. (2015). Optimal topology of reactive muffler achieving target transmission loss values: Design and experiment. Appl. Acoust..

[7] Altabey W.A. (2020). An exact solution for acoustic simulation based transmission loss optimization of double-chamber silencer. Sound Vib..

[8] Sastry J.S., Munjal M.L. (1995). A transfer matrix approach for evaluation of the response of a multi-layer infinite plate to a two-dimensional pressure excitation. Sound Vib..

[9] Beranek L.L., Mellow T. (2012). Acoustics: Sound Fields and Transducers.

[10] Reixach R., Del Rey R., Alba J., Arbat G., Espinach F.X., Mutjé P. (2015). Acoustic properties of agroforestry waste orange pruning fibers reinforced polypropylene composites as an alternative to laminated gypsum boards. Constr. Build. Mater..

[11] Tsai Y.T., Pawar S.J., Huang J.H. (2015). Optimizing material properties of composite plates for sound transmission problem. Sound Vib..

[12] Peat K.S., Rathi K.L. (1995). A finite element analysis of the convected acoustic wave motion in dissipative silencers. Sound Vib..

[13] Ji Z.L. (2005). Acoustic attenuation performance analysis of multi-chamber reactive silencers. Sound Vib..

[14] Ji Z.L. (2006). Boundary element analysis of straight-through hybrid silencer. Sound Vib..

[15] Levine H., Schwinger J. (1948). On the radiation of sound from an unflanged circular pipe. Phys. Rev..

[16] Norris A.N., Sheng I.C. (1989). Acoustic Radiation from a circular pipe with an infinite flange. J. Sound Vib..

[17] Silva F., Guillemain P., Kergomard J., Mallaroni B., Norris A.N. (2009). Approximation formulae for the acoustic radiation impedance of a cylindrical pipe. J. Sound Vib..

[18] Polack J.D., Meynial X., Kergomard J., Cosnard C., Bruneau M. (1987). Reflection function of a plane sound wave in a cylindrical tube. Rev. Phys. Appl..

[19] Dalmont J.P. (2001). Acoustic impedance measurement, part I: A review. J. Sound Vib..

[20] Dalmont J.P., Nederveen C.J., Joly N. (2001). Radiation impedance of tubes with different flanges: Numerical and experimental investigations. J. Sound Vib..

[21] Huang L. (2004). Parametric study of a drum-like silencer. J. Sound Vib..

[22] Barbieri R., Barbieri N. (2006). Finite element acoustic simulation based shape optimization of a muffler. Appl. Acoust..

[23] Barbieri R., Barbieri N. (2012). The technique of active/inactive finite elements for the analysis and optimization of acoustical chambers. Appl. Acoust..

[24] Lee S., Bolton J.S., Martinson P.A. (2016). Design of multi-chamber cylindrical silencers with microperforated elements. Noise Control Eng..

[25] Munjal M.L. (2020). Tuning a Two-Chamber Muffler for Wide-Band Transmission Loss. Int. J. Acoust. Vib..

[26] Lee J.K., Oh S.K., Lee J.W. (2020). Methods for evaluating in-duct noise attenuation performance in a muffler design problem. Sound Vib..

[27] Shi X.F., Mak C.M. (2016). Sound attenuation of a periodic array of micro-perforated tube mufflers. Appl. Acoust..

[28] Du T., Lee S.Y., Liu J.T., Wu D.Z. (2015). Acoustic performance of a water muffler. Noise Control Eng..

[29] Altabey W.A. (2022). The Damage Identification in Laminated Composite Plate under Fatigue Load through Wavelet Packet Energy Curvature Difference Method. Compos. Part C Open Access.

[30] Selameta A., Deniab F.D., Besa A.J. (2003). Acoustic behavior of circular dual-chamber mufflers. Sound Vib..

[31] Sheng M., Wang M., Sun J. (2001). The Basis of Noise and Vibration Control Technology.

[32] Selamet A., Ji Z.L. (1999). Acoustic attenuation performance of circular expansion chambers with extended inlet/outlet. Sound Vib..

[33] Altabey W.A. (2021). Applying deep learning and wavelet transform for predicting the vibration behavior in variable thickness skew composite plates with intermediate elastic support. J. Vibroeng..

[34] Li Z., Noori M., Wan C., Yu B., Wang B., Altabey W.A. (2022). A Deep Learning-Based Approach for the Identification of a Multi-Parameter BWBN Model. Appl. Sci..

[35] Ramachandran P., Zoph B., Quoc V.L. (2017). Searching for Activation Functions. arXiv.

[36] Noori M., Altabey W.A. (2022). Hysteresis in Engineering Systems. Appl. Sci..

[37] Altabey W.A., Noori M., Wang T., Ghiasi R., Kuok S.-C., Wu Z. (2021). Deep learning-based crack identification for steel pipelines by extracting features from 3d shadow modeling. Appl. Sci..

[38] Olah C. (2015). Understanding LSTM Networks. https://colah.github.io/posts/2015-08-Understanding-LSTMs/.

[39] Wang T., Li H., Noori M., Ghiasi R., Kuok S.-C., Altabey W.A. (2022). Probabilistic Seismic Response Prediction of Three-Dimensional Structures Based on Bayesian Convolutional Neural Network. Sensors.

[40] Zachary C.L., John B., Charles E. (2015). A Critical Review of Recurrent Neural Networks for Sequence Learning. arXiv.

[41] Hutter F., Hoos H.H., Leyton-Brown K. (2011). Sequential model-based optimization for general algorithm configuration. Learning and Intelligent Optimization.

[42] Diederik P.K., Jimmy B. (2014). Adam: A Method for Stochastic Optimization. arXiv.

[43] Martinez-Cantin R. (2014). Bayesopt: A bayesian optimization library for nonlinear optimization, experimental design and bandits. Mach. Learn. Res..

[44] Mohebian P., Aval S.B.B., Noori M., Lu N., Altabey W.A. (2022). Visible Particle Series Search Algorithm and Its Application in Structural Damage Identification. Sensors.

[45] Bergstra J., Bardenet R., Bengio Y., Kégl B. Algorithms for hyper-parameter optimization. Proceedings of the Advances in Neural Information Processing Systems.

[46] Bergstra J., Yamins D., Cox D. Making a science of model search: Hyperparameter optimization in hundreds of dimensions for vision architectures. Proceedings of the 30th International Conference on Machine Learning.

[47] Liu H., Lin J., Hua R., Dong L. (2022). Structural Optimization of a Muffler for a Marine Pumping System Based on Numerical Calculation. J. Mar. Sci. Eng..

